# Relationship Between Diet, Tinnitus, and Hearing Difficulties

**DOI:** 10.1097/AUD.0000000000000765

**Published:** 2020-02-24

**Authors:** Piers Dawes, Karen J. Cruickshanks, Antonia Marsden, David R. Moore, Kevin J. Munro

**Affiliations:** 1 Manchester Centre for Audiology and Deafness, School of Health Sciences, University of Manchester, Manchester, UK; 2 Manchester University Hospitals NHS Foundation Trust, Manchester Academic Health Science Centre, Manchester, UK; 3 Population Health Sciences, School of Medicine and Public Health, University of Wisconsin-Madison, Madison, WI, USA; 4 School of Health Sciences, Faculty of Biology, Medicine and Health, University of Manchester, Manchester, UK; 5 Cincinnati Children’s Hospital Medical Center, Cincinnati, OH, USA.

**Keywords:** Diet, Dietary pattern, Hearing difficulties, Nutrients, Presbyacusis, Tinnitus

## Abstract

**Objectives:**

Diet may affect susceptibility of the inner ear to noise and age-related effects that lead to tinnitus and hearing loss. This study used complementary single nutrient and dietary pattern analysis based on statistical grouping of usual dietary intake in a cross-sectional analysis of tinnitus and hearing difficulties in a large population study sample.

**Design:**

The research was conducted using the UK Biobank resource. Tinnitus was based on report of ringing or buzzing in one or both ears that lasts more than five minutes at a time and is currently experienced at least some of the time. Identification of a hearing problem was based on self-reported difficulties with hearing. Usual dietary intake and dietary patterns (involving statistical grouping of intake to account for how foods are combined in real-life diets) were estimated based on between two and five administrations of the Oxford Web-Q 24-hour dietary recall questionnaire over the course of a year for 34,576 UK adult participants aged 40 to 69.

**Results:**

In a multivariate model, higher intake of vitamin B12 was associated with reduced odds of tinnitus, while higher intakes of calcium, iron, and fat were associated with increased odds (B12, odds ratio [OR] 0.85, 95% confidence interval [CI] 0.75 to 0.97; Calcium, OR 1.20, 95% CI 1.08 to 1.34; Iron, OR 1.20, 95% CI 1.05 to 1.37; Fat, OR 1.33, 95% CI 1.09 to 1.62, respectively, for quintile 5 versus quintile 1). A dietary pattern characterised by high protein intake was associated with reduced odds of tinnitus (OR 0.90, 95% CI 0.82 to 0.99 for quintile 5 versus quintile 1). Higher vitamin D intake was associated with reduced odds of hearing difficulties (OR 0.90, 95% CI 0.81 to 1.00 for quintile 5 versus quintile 1), as were dietary patterns high in fruit and vegetables and meat and low in fat (Prudent diet: OR 0.89, 95% CI 0.83 to 0.96; High protein: OR 0.88, 95% CI 0.82 to 0.95; High fat: OR 1.16, 95% CI 1.08 to 1.24, respectively, for quintile 5 versus quintile 1).

**Conclusions:**

There were associations between both single nutrients and dietary patterns with tinnitus and hearing difficulties. Although the size of the associations was small, universal exposure for dietary factors indicates that there may be a substantial impact of diet on levels of tinnitus and hearing difficulties in the population. This study showed that dietary factors might be important for hearing health.

## Abstract 2

Supplemental Digital Content is available in the text.

## INTRODUCTION

Tinnitus refers to the perception of sound without an external source. Prevalence of tinnitus among UK adults aged 40 to 69 years was estimated at 16.9% (Dawes et al. 2014). Hearing impairment is common, affecting 36.7% of UK adults aged 61 to 70 years (defined as mean hearing threshold level of >25 dB hearing level over 500 to 4000 Hz in the better ear; Davis 1989). Encouragingly, there is evidence that both hearing loss and tinnitus may be associated with modifiable lifestyle factors including noise exposure, smoking, alcohol consumption, exercise, and diet, offering possibilities for prevention (Hoffman & Reed 2004; Cruickshanks et al. 2010). Diet may impact on susceptibility of the inner ear to noise and age-related effects that lead to hearing loss and tinnitus (Spankovich 2015).

### Diet and Tinnitus

The role of diet in tinnitus has been identified as a research priority by both patients and clinicians (Hall et al. 2013).There is anecdotal evidence for certain foods, single nutrients, and dietary supplements exacerbating or reducing tinnitus in individuals; however, there is little or no research evidence for a role of any particular dietary factor in contributing to tinnitus (British Tinnitus Association 2017; Seidman & Babu 2003; Patterson & Balough 2006). Limited research in relation to vitamin B12 and B3 and zinc deficiency and the effects of supplementation and tinnitus is inconsistent (Gersdorff et al. 1987; Hulshof & Vermeij 1987; Paaske et al. 1991; Shemesh et al. 1993; Ochi et al. 1997; Yetiser et al. 2002). Two small controlled studies reported a reduction in tinnitus among people with tinnitus and hyperinsulinemia following a diabetic diet rich in nutrients and low in fat and calories (Basut et al. 2003; Lavinsky et al. 2004). Only three population studies of dietary factors and tinnitus have been conducted to our knowledge (McCormack et al. 2014; Spankovich et al. 2017; Lee & Kim 2018). McCormack et al. (2014) reported inconsistent associations between intake of foods (based on an unvalidated set of quantitative food frequency questions) and persistent, bothersome, or transient tinnitus in a previous cross-sectional analysis of data from UK Biobank. Persistent tinnitus was associated with higher fruit and vegetable, bread, fish, and egg intake. Dairy and caffeinated coffee intake was associated with reduced odds of persistent tinnitus. Glicksman et al. (2014) also reported that higher caffeine intake was associated with lower risk of incident tinnitus in women. Spankovich et al. (2017) reported that a healthier diet (indexed by Healthy Eating Index [HEI] score; United States Department of Agriculture 1995) was associated with reduced odds of reported persistent tinnitus in cross-sectional analysis. Healthier HEI scores are characterized by higher intake of fruit and vegetables and whole grains, and so the associations observed by Spankovich et al. contradict McCormack et al.’s study. Lee and Kim (2018) reported that lower intakes of vitamin B2 and B3, protein, and water estimated from an unvalidated semiquantitative food frequency questionnaire were associated with tinnitus and tinnitus annoyance in a Korean population sample.

Taken together, these studies suggest a small but significant impact of diet on tinnitus in the general population. Spankovich et al. (2017) recommended further analysis of diet and tinnitus based on alternative diet models to the HEI and food- and nutrient-based analysis. The present study examines associations between tinnitus and nutrient intakes and dietary patterns using a validated 24-hr recall dietary questionnaire in a large population study sample.

### Diet and Hearing

The literature on diet and hearing in humans has been reviewed in detail elsewhere (Spankovich & Le Prell 2013; Spankovich 2015). Most previous research in diet and hearing has focused on single nutrient analysis. One advantage of single nutrient analysis is that many foods contribute to the intake of a particular nutrient, so nutrient-based analyses may be less susceptible to confounding with other behaviors than food-based analyses. The limitations are that nutrient intakes correlate with each other, so it may be difficult to isolate the effect of one nutrient from other. Single nutrient analysis does not take into account the likelihood of biochemical interactions between nutrients. Further, examining a wide range of single nutrients increases the likelihood of false-positive associations.

Despite the limitations of single nutrient analyses, some general conclusions about single nutrient intake and hearing may be possible. Higher intake of lipids, carbohydrates, and sugars is typically associated with poorer hearing. In recent studies, polyunsaturated fats were associated with better hearing, while high-density lipoprotein and triglycerides were associated with poorer audiometric hearing (Suzuki et al. 2000; Evans et al. 2006; Spankovich et al. 2011) and incident audiometric hearing loss (Dullemeijer et al. 2010; Gopinath, Flood, Rochtchina, et al. 2010a; Gopinath, Flood, Teber, et al. 2011) and incident self-reported hearing loss (Curhan et al. 2014). In relation to carbohydrates and sugars, Gopinath et al. (2010a) reported that higher glycemic index and glycemic load were associated with prevalent and incident audiometric hearing loss.

With respect to micronutrients, inconsistent associations between vitamins A, B, C, and E, and magnesium, and hearing have been reported (Houston et al. 1999; Gok et al. 2004; Durga et al. 2007; Michikawa et al. 2009; Gopinath, Flood, Rochtchina, et al. 2010b; Shargorodsky et al. 2010; Gopinath, Flood, McMahon, et al. 2011; Spankovich et al. 2011; Choi et al. 2014; Kang et al. 2014; Curhan et al. 2015). Besides the possibility of false-positive results described earlier, the inconsistency of single nutrient research may be attributable to differences in study design (e.g., cross-sectional versus longitudinal), hearing measures (e.g., self-reported versus audiometric), and dietary measures (e.g., questionnaire versus serum-based).

An alternative and complementary approach to single nutrient analysis is dietary pattern analysis (Hu 2002), which involves statistically grouping diets together (based on nutrients, foods, or food groups) or describing dietary patterns with reference to recommended dietary intake. Dietary patterns account for the combined effect of various foods, for interactions between nutrients, and describe how foods are combined in real-life diets. An entire diet may have a stronger association with disease risk than for individual components (Gao et al. 2007).

We are aware of two studies that have examined dietary patterns in relation to hearing. Spankovich and LePrell (2013) carried out cross-sectional analysis of data from the National Health and Nutritional Examination Survey. Higher dietary quality (based on mapping dietary intake to the US HEI; United States Department of Agriculture 1995) was associated with better high-frequency hearing. There was a significant interaction with noise exposure history, with greater noise exposure and poor diet being associated with poor hearing (Spankovich & Le Prell 2014). Curhan and colleagues (2018) reported that healthier diets (indexed by Alternate Mediterranean diet [Fung et al. 2009] and Dietary Approaches to Stop Hypertension [Vollmer et al. 2001] scores but not HEI scores) were associated with reduced risk of incident self-reported hearing loss among women. The primary disadvantage with describing dietary patterns with reference to an “ideal” diet such as the HEI is that ideal diets recommended in national guidelines vary. There is no empirical basis for choosing one dietary guideline over another or for supposing that the dietary guideline in question is ideal in terms of hearing health. The alternative to mapping dietary intake onto dietary guidelines is to carry out an analysis of dietary patterns within the population based on actual food or nutrient intake (Hu 2002), as was done in the present study. The present study of hearing impairment and tinnitus uses complementary nutrient and dietary pattern analysis based on statistical analysis of usual dietary intake in a large and inclusive sample.

## MATERIALS AND METHODS

The analysis was conducted using the UK Biobank resource (Collins 2012). UK Biobank is an international resource for health research and contains data from over 500,000 UK adults aged 40 to 69 years at the time of initial assessment. The UK Biobank sample is not representative of the UK general population; however, the disease–exposure relationships are thought to be generalizable due to the size and inclusiveness of the sample (Fry et al. 2017). The Web-Q diet questionnaire (described later) was introduced into the UK Biobank assessment protocol towards the end of data collection for the last 70,000 participants. Recruitment to the UK Biobank was via the UK National Health Service and aimed to be as inclusive as possible of the UK population. Recruitment was via a postal invitation with a telephone follow-up. The response rate was 5.47%. Participants were tested between 2006 and 2010. Participants attended a UK Biobank assessment centre and gave informed written consent. Participants completed a “whole body” assessment of 90 min duration that included a computerized questionnaire on medical history, lifestyle and environment and physical measures. Detailed information of the testing procedure and additional data collected can be found elsewhere (http://www.ukbiobank.ac.uk/).

Data on sex, race (based on 2001 UK Census categories), and area of residence were recorded for each participant. Area of residence was translated to Townsend deprivation score, a proxy measure of socioeconomic status (Norman 2010). Townsend scores are widely used in health studies. Scores are based on four variables including unemployment, noncar ownership, nonhome ownership, and household overcrowding. Each variable is expressed as a *z* score relative to national levels that are then summed to provide an overall deprivation score. Lower scores represent less deprived (more affluent) socioeconomic status. In the analyses described later, Townsend scores were categorized into quartiles from least to most deprived sections of the study sample. Race was coded according to “White” or “Nonwhite” background.

A subset of the UK Biobank was included in the present analysis. Participants were excluded from analysis on the basis of (i) outside age range of 40 to 69 years, (ii) no self-report hearing or tinnitus data, (iii) estimated energy intake <597 or >4300 kcal/day or >5 SD above or below the mean, (iv) having completed less than two instances of the 24-hr dietary recall questionnaire, (v) estimated dietary intake more than 5 SDs above the mean for any nutrient, and (vi) self-reported use of a dietary supplement at each instance of completion of the dietary recall questionnaire. At least two instances of completion of the dietary recall questionnaire were required for estimation of usual dietary intake (Harttig et al. 2011). Regular users of dietary supplements (i.e., vitamin and mineral tablets or tonics, as indicated by self-reported use of a dietary supplement at each instance of dietary assessment) were excluded because the dietary assessment method (Web-Q- see later) did not account for nutrient intake from dietary supplements. Non- or occasional users of supplements were included on the assumption that usual nutrient intake would primarily be determined by intake of food/beverages.

### Hearing and Tinnitus

Identification of a hearing problem was based on a response of “yes” to the question “Do you have any difficulty with your hearing?”. Estimates of the accuracy of self-report vary according to the level of hearing impairment, though they are typically around 90% sensitivity with 70% specificity for moderate levels of hearing impairment (Clark et al. 1991; Nondahl et al. 1998; Sindhusake et al. 2001). Identification of tinnitus was based on responses to the question “Do you get or have you had noises (such as ringing or buzzing) in your head, or in one or both ears, that lasts for more than five minutes at a time?”. Tinnitus was identified based on responses of “yes most of the time,” “yes a lot of the time,” or “yes some of the time.” There is no established objective measure of tinnitus, and tinnitus is typically measured using self-report. This tinnitus measure in this study is similar to those used in other studies of the epidemiology of tinnitus (Davis 1989; Davis & Rafaie 2000; Gopinath, McMahon, Rochtchina, et al. 2010).

### Diet

Dietary assessment was based on the Oxford Web-Q, a detailed computerized questionnaire on the intake of 200 commonly consumed food and beverages consumed in the previous 24 hr (Liu et al. 2011). The Web-Q takes less than 15 min to complete, and data are comparable to traditional interviewer-administered 24-hr dietary recall questionnaires (Liu et al. 2011). Mean correlation between interviewer-administered questionnaire and Web-Q nutrient estimates was 0.6 with the majority between 0.5 and 0.9. Mean differences in estimated intake were less than 10% for all nutrients except for vitamin B12 and D, where the Web-Q underestimated intake versus interviewer-administered questionnaire (Liu et al. 2011). Participants are presented with a yes/no question (e.g., did you eat any bread or crackers yesterday?). Positive answers result in an expanding selection of additional questions. Participants were required to select the amount of each food consumed using standard serving categories or portions (Ministry of Agriculture Fisheries and Food 1993). Descriptions of serving sizes for each food were available via a “help” option. Participants who had provided UK Biobank with an email contact address were invited to complete the questionnaire four additional times over the course of one year in order to account for seasonal variation in dietary intake and provide an estimate of habitual intake for each individual. Email invitations were sent on a range of days of the week in order to capture variation in diet between weekdays and weekend days. Participants were allowed up to three days to complete the questionnaire for the first three rounds of invitations. Participants were allowed 14 days for the final two rounds of invitations. The Web-Q automatically calculated estimates of vitamin and mineral intake (see Table 1 for the nutrient parameters) for each participant for each occasion of assessment with the Web-Q based on multiplying the amount of the food/beverage consumed by the nutrient composition of the food/beverage (Food Standards Agency 2002). Use of vitamin and mineral supplements was recorded but was not used in the estimation of daily nutrient intake values. In the present study, usual dietary intake was estimated from at least two instances of the Web-Q questionnaire using the Multiple Source Method (Harttig et al. 2011), a statistical method for estimating usual dietary intake on the basis of two or more short-term measurements (e.g., 24-hr dietary recall).

**TABLE 1.**
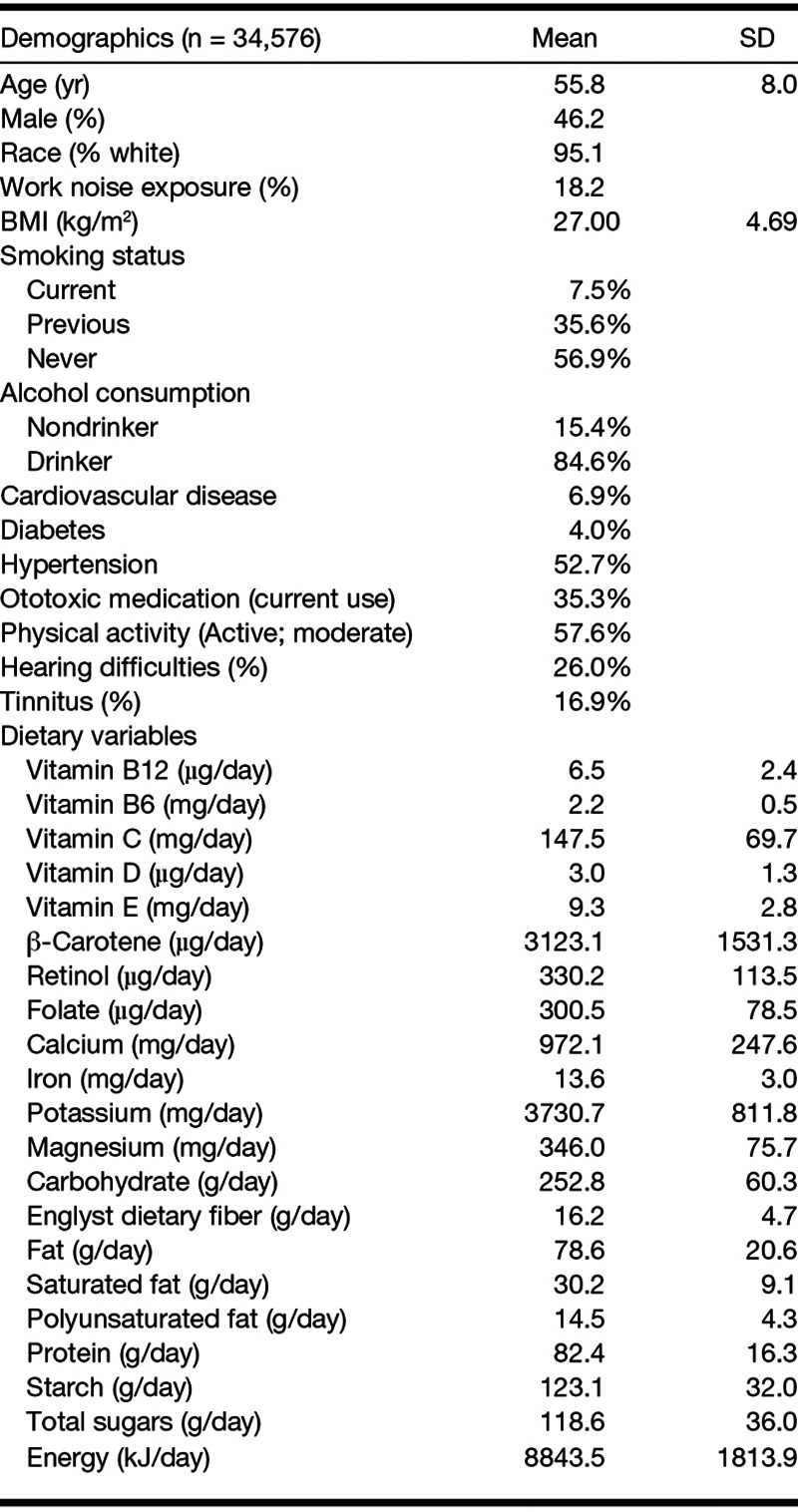
Characteristics of participants in the study sample according to demographic variables and estimated nutrient intake

### Cardiovascular Disease, Cholesterol, Hypertension, and Diabetes

Cardiovascular disease was identified if a participant reported any cardiovascular problem including heart attack, heart failure, stroke, angina, intermittent claudication, transient ischemic attack, and arterial embolism or deep venous thrombosis. High cholesterol was identified on the basis of participant report of high cholesterol or if the participant reported they were currently taking medication for high cholesterol. Hypertension was identified on the basis of participant report of high blood pressure, currently took medication for high blood pressure, or a measured systolic blood pressure greater than 140 mm Hg or diastolic pressure greater than 90 mm Hg. Diabetes was identified on the basis of participant report of diabetes or the use of medication for diabetes.

### Body Mass Index, Ototoxic Medication, Occupation- and Music-Related Noise Exposure, Physical Activity, Smoking, and Alcohol Consumption

Body mass index (BMI) was calculated as the participants’ weight (in kilograms) divided by height squared (in meters). All medications that were being taken regularly (daily, weekly, or monthly) were recorded. Short-term medications (e.g., a short course of antibiotics) were not recorded. All medications known to have ototoxic properties (including loop diuretics, aminoglycoside antibiotics, quinine derivatives, nonsteroidal anti-inflammatories, and salicylates) were coded as such. Occupation-related noise exposure was identified on the basis of any reported noise exposure in response to the question “Have you ever worked in a noisy place where you had to shout to be heard?” Music-related noise exposure was identified on the basis of any reported exposure in response to the question “Have you ever listened to music for more than 3 hr per week at a volume which you would need to shout to be heard or, if wearing headphones, someone else would need to shout for you to hear them?” The criterion for work- and music-related noise roughly corresponds to exposure exceeding 85 dB(A) (Health and Safety Executive 1989). Participants were classified as “inactive” if they reported doing less than 10 min or no physical activity in response to the question “Yesterday, about how long did you spend doing activities that needed moderate effort, making you somewhat short of breath? For example walking upstairs, going to the gym, jogging, energetic dancing, aerobics, most sports, using heavy power tools and other physically demanding DIY & gardening.” Participants were classified as “active” if they reported doing more than 10 min of activity. Smoking status was described in response to two questions “Do you smoke tobacco now?” and “In the past, how often have you smoked tobacco?” Nonsmokers were those who reported never smoking or reported having tried smoking once or twice. Exsmokers were those who reported previously smoking on most or all days. Current smokers were those who reported currently smoking occasionally or on most or all days. Current alcohol consumption was described on the basis of responses to the question “About how often do you drink alcohol?” Drinkers were identified on the basis of any report of current alcohol consumption (including the response options “Special occasions only,” “One to three times a month,” “One or twice a week,” “Three or four times a week,” and “Daily or almost daily”). Nondrinkers were identified on the basis of a response of “Never.”

### Analysis

Both raw nutrient intake and nutrient intake adjusted for individual variation in energy intake were analyzed. Two methods were used to adjust for variation in individual energy intake (Willett 2013): (i) nutrient density: nutrients were adjusted for energy density (by dividing the average daily nutrient intake by the average daily energy intake multiplied by 1000 to provide an estimate of nutrient intake per 1000 kcal) and (ii) multivariate nutrient density including total energy intake in a multivariate model along with the nutrient density estimates. The analysis presented in the present study was based on nutrient density values, because this method is commonly used in nutritional epidemiology and because nutrient density values are used by national dietary guidelines. Analyses with alternative unadjusted and adjusted nutrient data gave similar results. Dietary pattern analysis was based on principal components analysis of estimates of usual nutrient intake (nutrient density values) to identify underlying patterns of food/beverage consumption (Hu 2002). Principal components analysis was carried out with varimax rotation, with factors with eigenvalues greater than 1 extracted.

Logistic regression analyses were carried out to model cross-sectional associations between micro- and macronutrients and dietary patterns with hearing difficulties and tinnitus. Nutrients and dietary pattern components were entered as quintile data. Classification of dietary intake into quintiles is commonly used in dietary epidemiology because analysis by quintiles of intake does not require the assumption of a linear dose–response relationship and reduces the impact of outlying data points. Quintile 1 (the lowest level of intake) was the reference category in the following analyses. All nutrient/dietary components were entered simultaneously into the regression models. Potential confounders were identified based on having been implicated with hearing loss or tinnitus in previous research (Cruickshanks et al. 2010; Lin et al. 2011; Lu et al. 2012) and checked for associations with the dietary patterns calculated later. Initial models were run with each nutrient/dietary component and all possible confounders (including age, sex, smoking, diabetes, ototoxic medication, cardiovascular disease, work noise exposure, music noise exposure, and BMI). The main model for tinnitus did not adjust for hearing and vice versa as the two outcomes are themselves associated, and each may be on the causal pathway to the other. However, to establish whether associations between dietary factors and tinnitus were independent of hearing and vice versa, models were rerun including hearing and tinnitus as covariates in the analyses for tinnitus and hearing, respectively.

## RESULTS

There were 47,072 participants who had completed at least two instances of the Web-Q and hearing and tinnitus questions. Participants who were regular supplement users (those who reported using a dietary supplement at every instance of the Web-Q; n = 12,496) were excluded. Table 1 shows the characteristics of the 34,576 participants from the UK Biobank that fit the criteria for inclusion according to demographic variables, alcohol consumption, smoking status, hearing difficulties, tinnitus and covariates, and estimates of nutrient intake. Prevalence of self-reported hearing difficulties was 26%, and 16.9% reported tinnitus. In total, 9.2% reported both tinnitus and hearing difficulties.

With respect to completion of the Web-Q, 10% (3451) of participants completed the Web-Q five times, 24% (8327) four, 31% (10,683) three, and 25% (12,115) on two occasions. There was representation of each season of the year (Spring 25%; Summer 34.5%; Autumn 29.3%; and in Winter 11.6% of questionnaires) and day of the week (Monday 17%; Tuesday 15.8%; Wednesday 14.8%; Thursday 14.7%; Friday 14.2%; Saturday 12.5%; Sunday 10.9% of questionnaires).

Principal components analysis yielded six components with Eigenvalues greater than 1. The six components accounted for 78% of variation in diet, following varimax rotation (Table 2). To assist with interpretation of the factors, correlations between each factor and nutrient with an absolute value greater than 0.5 are in bold font.

**TABLE 2.**
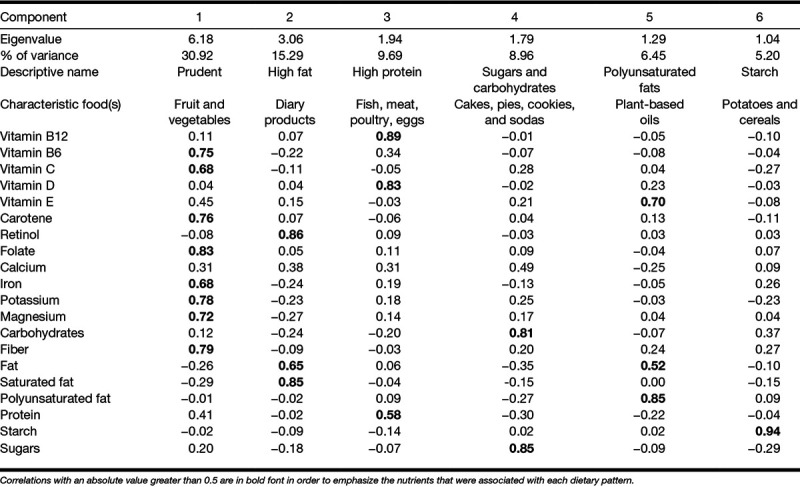
Dietary pattern analysis: correlation matrix

### Tinnitus

The final regression models for tinnitus included age, sex, race, ototoxic medication, occupation-related noise exposure, smoking status, alcohol consumption, and cardiovascular disease. Higher intake of vitamin B12 and dietary pattern factor 3 (High protein) was associated with reduced odds of tinnitus (Table 3). Higher intakes of calcium, iron, and fat were associated with increased odds of tinnitus. Adding hearing as a covariate to the regression model made little difference to the pattern of associations observed (See Table 1 in Supplemental Digital Content 1, http://links.lww.com/EANDH/A550).

**TABLE 3.**
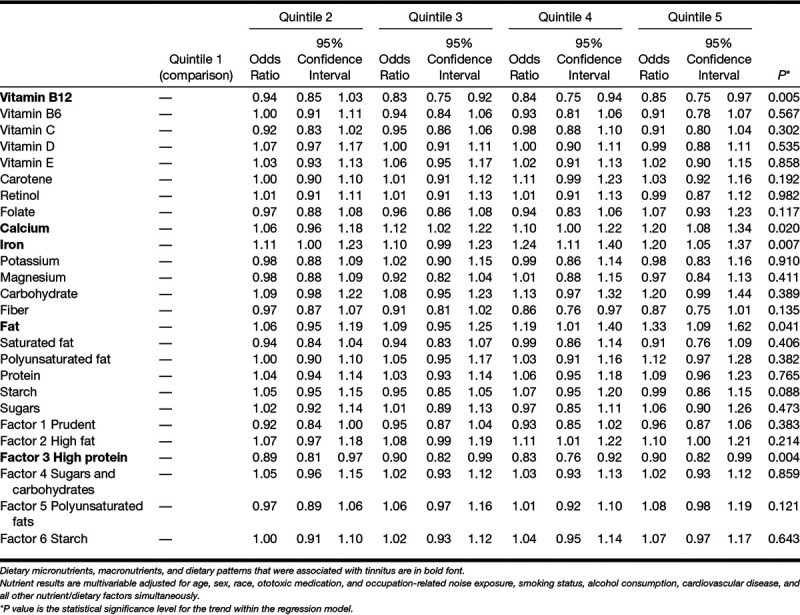
Tinnitus: odds ratios for dietary micronutrients, macronutrients, and dietary patterns by quintile

### Hearing Difficulties

The final regression models for hearing included age, sex, race, ototoxic medication, BMI, occupation-related noise exposure, smoking status, alcohol consumption, physical activity, and hypertension. Higher intake of fat and saturated fat was associated with higher odds of reporting hearing difficulties (Table 4). Higher intake vitamin D, dietary factor 1 (Prudent), and factor 3 (High protein) were associated with reduced odds of hearing difficulties. Factor 2, a dietary pattern associated with high fat intake, was associated with higher odds of hearing difficulties. Rerunning the analysis with the addition of tinnitus, the pattern of associations with hearing was similar apart from vitamin B12 (with higher intakes associated with poorer hearing; OR 1.17, 95% confidence interval 1.04 to 1.31; for quintile 5 versus quintile 1) and factor 3 High-protein dietary pattern (which was no longer associated with hearing; see Table 2 in Supplemental Digital Content 2, http://links.lww.com/EANDH/A551).

**TABLE 4.**
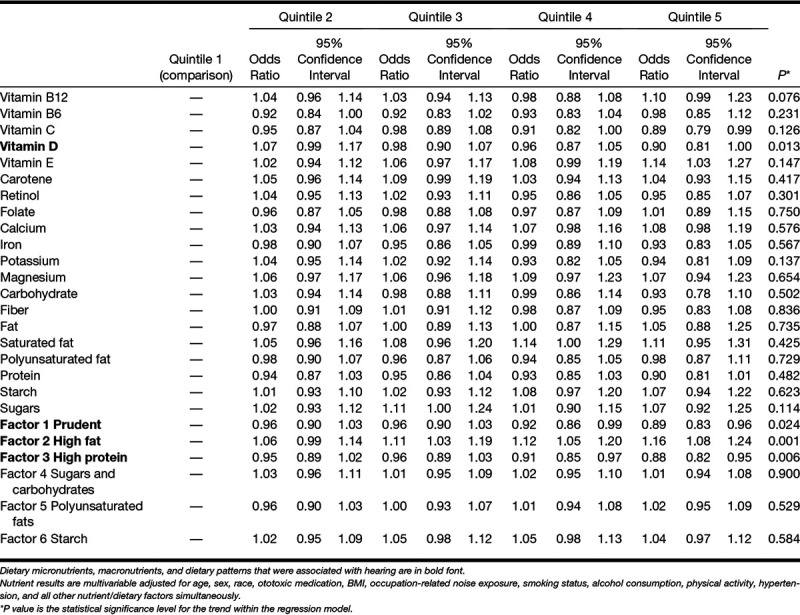
Hearing: odds ratios for dietary micronutrients, macronutrients, and dietary patterns by quintile

There were no interactions between age, sex, BMI or race and B12, calcium, iron, fat or factor 3 dietary intakes and tinnitus. There were no interactions between age, sex, BMI or race and vitamin D, factor 1, 2, or 3.

Around one-third of UK Biobank participants completed a test of speech recognition in noise (Smits et al. 2004). Patterns of association with a hearing phenotype based on performance of the speech test were similar to those observed with the larger cohort of participants who had completed the self-reported hearing measure.

All analyses were rerun including the 11,008 participants who were excluded from the analysis earlier having been identified as regular users of dietary supplements. A similar pattern of results was obtained for both tinnitus and hearing difficulties.

## DISCUSSION

This study reports evidence of associations between both single nutrients and dietary patterns with tinnitus and hearing difficulties. The size of the associations was generally small, but the universal exposure that the dietary data represent implies there may be a substantial impact of diet on levels of tinnitus and hearing difficulties within the population.

### Tinnitus

Higher intakes of calcium, iron and fat were associated with increased odds of tinnitus while higher intakes of vitamin B12 and dietary pattern factor 3 (High protein) were associated with reduced odds of tinnitus. Adjustment for hearing difficulties made no difference to patterns of associations between tinnitus and dietary factors, suggesting that possible impacts of diet on tinnitus are independent of hearing.

The present study is the only study we are aware of to suggest a specific association between levels of dietary B12 and tinnitus. The association between factor 3 (High protein) intake and reduced odds of tinnitus may be related to vitamin B12; meat, fish, and poultry are sources of vitamin B12. A recent Korean population study similarly reported that lower intakes of protein, vitamin B2, and vitamin B3 were associated with increased tinnitus (Lee & Kim 2018). One study reported vitamin B12 deficiency in a sample of soldiers with tinnitus and noise-induced hearing loss compared with a control group with hearing loss only (Shemesh et al. 1993). The specific mechanism of action is unclear (Patterson & Balough 2006). Unfortunately, the likely etiology and any further detail about the nature of the tinnitus were not available in the present study. Future studies may investigate whether dietary intake or supplementation of vitamin B12 is associated with reduced tinnitus when it occurs in subgroups such as following noise exposure. Vitamin B12 would be best examined using blood biomarkers rather than indices of dietary intake because of variation of absorption of B12 (Green 2011).

We previously reported that “persistent tinnitus” was associated with higher fruit and vegetable, bread, fish, and egg intake. Dairy and caffeinated coffee intake was associated with reduced odds of persistent tinnitus (McCormack et al. 2014). “Bothersome tinnitus” was associated with higher intake of wholemeal/wholegrain bread. Reduced odds of “transient tinnitus” were associated with dairy, caffeinated coffee, and bread intake. The association between B12 intake and tinnitus in the present study seems consistent with some previous results (e.g., dairy linked with reduced risk of tinnitus; dairy is a source of vitamin B12), while others are not (egg intake linked with increased risk of tinnitus; eggs are also a source of vitamin B12). We argue that the present analysis is an improvement on our previous analysis because (i) nutrient intakes were estimated from multiple instances of a validated 24-hr dietary recall questionnaire rather than responses to an unvalidated set of food frequency questions assessed at only one time point and (ii) tinnitus categories in our previous article are difficult to interpret; perception of tinnitus as being “bothersome” or not may depend on psychological or personality variables, not just severity (McCormack et al. 2015). It is unclear how psychological variables might interact with diet on the experience of tinnitus. A single tinnitus phenotype was reported in the present study, based on a definition that is commonly used in epidemiology of tinnitus.

Higher intake of fat and starch was associated with increased risk of tinnitus. Spankovich et al. (2017) also reported that an unhealthy diet characterized by high fat and low fruit and vegetable intake was associated with increased tinnitus. Spankovich did not offer a causal explanation for the impact of high fat diets on tinnitus. High saturated fat intake has previously been linked to hearing loss possibly via cardiovascular disease pathways (Suzuki et al. 2000; Evans et al. 2006; Gopinath, Flood, McMahon, et al. 2010; Spankovich et al. 2011). Increased risk of tinnitus associated with fat intake may also occur via cardiovascular pathways.

Abnormalities in the calcium signaling pathway in outer hair cells have previously been linked to tinnitus (Zenner & Ernst 1993; Sziklai 2004), and calcium channel blockers have been investigated as a treatment for tinnitus (Davies et al. 1994). Iron levels could conceivably impact hearing health via oxygen transport and oxygenation of the organs of hearing. However, Lee and Kim (2018) reported no association between dietary iron intake and tinnitus in a sample of middle-aged Korean adults. Further, higher intake of iron associated with increased odds of tinnitus seems contradictory to the finding of a high-protein diet linked to lower odds of tinnitus; meat, fish and poultry are sources of iron.

### Hearing Difficulties

Higher intake of vitamin D and dietary patterns characterized by high fruit and vegetable intake (factor 1) and high protein (factor 3) was associated with reduced odds of hearing difficulties. A dietary pattern associated with high saturated fat intake (factor 2) was associated with increased risk of hearing difficulties.

The association between a high-fat diet and poorer hearing is consistent with a growing body of research in relation to a diet high in saturated fats and poor hearing (Rosen & Olin 1965; Rosen et al. 1970; Suzuki et al. 2000; Evans et al. 2006; Dullemeijer et al. 2010; Gopinath, Flood, Rochtchina, et al. 2010a; Gopinath, Flood, Teber, et al. 2011; Spankovich et al. 2011). A diet low in saturated fat may be protective against hearing loss. Previous studies reported associations between higher intake of polyunsaturated fats with reduced risk of incident audiometrically identified hearing loss (Dullemeijer et al. 2010; Gopinath, Flood, Rochtchina, et al. 2010a) and incident self-reported hearing loss (Curhan et al. 2014). There was no association between intake of polyunsaturated fats and hearing difficulties in the present study, perhaps due to the cross-sectional design of the present study versus longitudinal designs of previous studies. Similarly, carbohydrate and sugar intake was not related to hearing though previous studies suggested that sugary high glycemic index nutrition may be linked to poorer audiometric hearing (Rosenhall et al. 2015) and incident audiometric hearing impairment (Gopinath, Flood, McMahon, et al. 2010).

Consistent with other dietary pattern analyses showing that healthy diets were associated with reduced risk of audiometric poor hearing (Spankovich & Le Prell 2013) and self-reported incident hearing loss (Curhan et al. 2018), dietary patterns that were high in fruit, vegetables, fish, poultry, and eggs were associated with lower odds of hearing difficulties. Healthy diets low in saturated fat may promote good hearing health.

Higher levels of vitamin D were associated with better hearing in the present study. The association between factor 3 (High protein) intake and better hearing may be related to vitamin D intake, as oily fish, red meat, and liver are sources of vitamin D. In contrast, a Korean population study (Kang et al. 2014) reported higher serum concentrations of vitamin D associated with *poorer* mid- and high-frequency audiometic hearing. Brookes (1983) reported several case studies of progressive sensorineural hearing loss in patients with vitamin D deficiency in the United Kingdom. In addition to dietary sources, vitamin D is synthesized in the skin with exposure to sun. Dietary sources of vitamin D may become more important when synthesis via sun exposure is not sufficient for physiological need (Stanbury & Mawer 1978), which may explain why dietary vitamin D was associated with better hearing in the sun-deprived northern European population that was the focus of the present study. Alternatively, population differences in responsiveness to UVB radiation (Binkley et al. 2007) and/or genetics of vitamin D metabolism (Wang et al. 2010) may account for differences in patterns of association observed between countries.

In models of dietary factors with hearing, addition of tinnitus as a covariate resulted in intake of B12 associated with *poorer* hearing while high-protein diet was no longer associated with hearing. Possible explanations are that tinnitus may mediate the impact of diet on hearing, the self-reported measures of hearing are confounded, or that one or both associations are spurious. Disentangling relationships between tinnitus, hearing, and dietary factors may require mediation analysis based on longitudinal observational data, followed by experimental studies with random allocation to dietary condition.

### Study Limitations

It was not possible to establish causal associations based on the cross-sectional correlational design on the present study or to examine the time course of exposure to dietary factors and development of hearing difficulties/tinnitus. The associations described in the present study may be due to confounding with other dietary or nondietary factors or to insufficient control of measured confounders. Following the precedent of previous single nutrient studies, the analyses were not adjusted for type-1 statistical errors; some of the statistically significant associations reported in the present study may be false positives. Encouragingly, the present study reported similar associations (e.g., in relation to high-fat diets and hearing) that have been reliably reported in previous independent studies. Additionally observational prospective cohort studies and/or intervention studies of dietary supplementation would provide more convincing evidence of causal associations.

Hearing was indexed with a self-reported measure rather than measured thresholds, although self-reported hearing difficulty is a reliable index of audiometrically identified hearing impairment (Nondahl et al. 1998), and almost all UK Biobank participants completed the item about self-reported hearing difficulties so that these data were available for everyone that had completed the dietary measure.

Nutrients were estimated from dietary intake rather than biomarkers; circulating levels are likely to differ from those estimated from intake due to homeostatic mechanisms, individual differences in absorption, bioavailability, errors in nutrient estimation based on food/beverage intake, and other factors (Van Dam & Hunter 2013). Dietary data were only available on a subset of the UK Biobank sample due to late addition of the measure. Different numbers of participants completed up to five instances of the dietary measure, and only 34% of participants completed the dietary measure at least four times. Completion of the dietary measure was reasonably equal across days of the week, although the proportion of dietary measures completed in winter was lower than in the other seasons. The implication of the low winter yield is that annual consumption of fresh fruit and vegetables may be overestimated.

One disadvantage of single nutrient approaches is that single nutrients are not consumed in isolation. Nutrient intakes correlate with each other, and single nutrient analysis does not take into account the possibility of biochemical interactions between nutrients. One approach to address the limitations of single nutrient analysis is to examine dietary patterns rather than single nutrients. This was the first study to use dietary pattern analysis based on statistical identification of dietary patterns within a population in relation to hearing and tinnitus. A focus on dietary patterns is complementary to the single nutrient approach that the majority of previous dietary hearing research used. The limitations of dietary pattern analyses are that dietary patterns identified from the data must be interpreted post hoc, similar diets may be named differently across studies, and dietary patterns identified in one population may not be generalizable to other populations in which dietary groupings may be different. The effects of single nutrients that do significantly affect health may be diluted and missed, and associations with overall dietary patterns may not inform the biological mechanism underlying disease.

The UK Biobank recorded use of dietary supplements, but the exact formulation of supplements was not recorded, and it was not possible to include intake from supplements or adjust for intake from supplements in the analysis. Users of dietary supplements differ from nonusers in various ways including health, demographics, lifestyle, and diet (Lyle et al. 1998). Encouragingly, analysis including regular supplement users yielded a similar pattern of results to the analysis that excluded regular supplement users. A useful test of the impact of specific nutrients on hearing and tinnitus would be to model the effect of supplementation on hearing/tinnitus risk with the expectation that supplementing specific dietary nutrients associated with reduction in hearing/tinnitus risk may result in further decreased risk. Where such data are available, modeling the impact of supplementation of specific nutrients on hearing/tinnitus may be informative.

Information on occupation- and music-related noise exposure was based on self-reported levels of noise exposure rather than direct measurement of noise levels experienced by participants. Some residual confounding with noise exposure may therefore be likely.

## CONCLUSIONS

This study suggested a substantial impact of diet on levels of tinnitus and hearing difficulties.

## SHORT SUMMARY

Dietary factors are thought to influence susceptibility to tinnitus and hearing loss. This study examines cross-sectional associations between tinnitus, hearing difficulties, and nutrient intakes and dietary patterns using a validated 24-hr recall dietary questionnaire in a large population study sample of 34,576 UK adults. Dietary nutrients and patterns were estimated based on repeated administrations of a questionnaire over one year. After adjusting for confounders, higher intakes of calcium, iron, and fat were associated with increased odds of tinnitus, while higher intakes of vitamin B12 and a dietary pattern high in meat intake were associated with reduced odds of tinnitus. Higher intake of vitamin D and diets high in fruit, vegetable, and meat were associated with reduced odds of hearing difficulties. A high-fat diet was associated with increased risk of hearing difficulties. The study suggests that a diet low in fat and high in vitamins and antioxidants may be important for hearing health.

## ACKNOWLEDGMENTS

The authors thank Avni Vayas for assisting with interpretation of the factors in the dietary pattern analysis.

## Supplementary Material


