# Supplementary material for: Relationship Between Diet, Tinnitus, and Hearing Difficulties
Source: Ear Hear. 2020 Feb 24;41(2):289–99. doi: 10.1097/AUD.0000000000000765 (PMC7664714; doi:10.1097/AUD.0000000000000765)
Supplement: Supplementary file 1 [file aud-41-289-s001.pdf]

Supplemental Table 1. Tinnitus: Odds ratios for dietary micronutrients, macronutrients and dietary patterns by quintile adjusting for hearing

	Quintile 1 (comparison)	Quintile 2 Odds Ratio	95% confidence interval		Quintile 3 Odds Ratio	95% confidence interval		Quintile 4 Odds Ratio	95% confidence interval		Quintile 5 Odds Ratio	95% confidence interval		*p* value*
**Vitamin B12**	**-**	0.93	0.83	1.03	0.82	0.74	0.92	0.83	0.74	0.94	0.81	0.71	0.93	0.006
Vitamin B6	-	1.03	0.92	1.15	0.95	0.84	1.08	0.93	0.81	1.07	0.90	0.76	1.07	0.456
Vitamin C	-	0.93	0.84	1.04	0.95	0.84	1.06	1.01	0.89	1.14	0.96	0.84	1.10	0.445
Vitamin D	-	1.03	0.93	1.14	1.01	0.91	1.13	1.00	0.89	1.12	1.01	0.89	1.15	0.980
Vitamin E	-	1.03	0.92	1.14	1.06	0.95	1.18	1.02	0.91	1.15	1.00	0.88	1.14	0.827
Carotene	-	0.99	0.89	1.10	0.96	0.86	1.07	1.10	0.98	1.24	1.00	0.88	1.14	0.077
Retinol	-	1.01	0.91	1.12	1.00	0.90	1.12	1.01	0.90	1.14	1.00	0.88	1.15	0.999
Folate	-	0.98	0.87	1.09	0.99	0.88	1.12	0.95	0.83	1.09	1.08	0.93	1.26	0.221
**Calcium**	-	1.04	0.93	1.16	1.08	0.98	1.19	1.07	0.96	1.18	1.21	1.07	1.36	0.029
**Iron**	**-**	1.13	1.01	1.26	1.12	1.00	1.26	1.28	1.13	1.45	1.23	1.06	1.42	0.004
Potassium	-	0.98	0.88	1.10	1.05	0.92	1.20	1.03	0.89	1.20	1.00	0.83	1.20	0.716
Magnesium	-	0.95	0.85	1.06	0.90	0.79	1.03	0.99	0.86	1.14	0.97	0.82	1.14	0.330
Carbohydrate	-	1.08	0.96	1.21	1.10	0.95	1.26	1.16	0.98	1.38	1.28	1.05	1.57	0.172
Fibre	-	0.98	0.88	1.10	0.91	0.81	1.03	0.86	0.75	0.99	0.90	0.76	1.05	0.157
**Fat**	-	1.08	0.96	1.22	1.12	0.97	1.30	1.22	1.03	1.45	1.38	1.12	1.71	0.036
Saturated Fat	-	0.95	0.84	1.06	0.94	0.82	1.07	0.98	0.84	1.15	0.90	0.74	1.09	0.517
Polyunsat. Fat	-	1.00	0.90	1.11	1.06	0.95	1.19	1.03	0.91	1.18	1.10	0.94	1.28	0.611
Protein	-	1.05	0.95	1.17	1.06	0.95	1.19	1.09	0.97	1.23	1.14	1.00	1.31	0.429
Starch	-	1.06	0.96	1.18	0.95	0.84	1.06	1.03	0.91	1.17	0.96	0.82	1.13	0.163
Sugars	-	0.99	0.89	1.11	0.95	0.83	1.08	0.92	0.79	1.07	1.01	0.84	1.21	0.404
Factor 1 Prudent	-	0.93	0.84	1.02	0.99	0.89	1.09	0.97	0.88	1.07	1.03	0.93	1.15	0.301
Factor 2 High fat	-	1.07	0.96	1.18	1.07	0.97	1.18	1.09	0.99	1.21	1.09	0.98	1.21	0.444

**Factor 3 Meat**	-	0.88	0.80	0.97	0.91	0.83	1.01	0.84	0.76	0.93	0.92	0.83	1.02	0.014
Factor 4 Sugars & carbohydrates	-	1.03	0.94	1.14	0.99	0.89	1.09	1.01	0.92	1.12	1.02	0.92	1.13	0.908
Factor 5 Polyunsat. fats	-	1.00	0.90	1.10	1.04	0.94	1.15	1.02	0.93	1.13	1.10	0.99	1.21	0.328
Factor 6 Starch	-	1.02	0.92	1.13	1.04	0.94	1.15	1.02	0.92	1.13	1.07	0.97	1.19	0.682

**p* value is the statistical significance level for the trend within the regression model.

Nutrient results are multivariable adjusted for age, sex, race, ototoxic medication, and occupation-related noise exposure, smoking status, alcohol consumption, cardiovascular disease, hearing difficulties and all other nutrient/dietary factors simultaneously.
