# Supplementary material for: Relationship Between Diet, Tinnitus, and Hearing Difficulties
Source: Ear Hear. 2020 Feb 24;41(2):289–99. doi: 10.1097/AUD.0000000000000765 (PMC7664714; doi:10.1097/AUD.0000000000000765)
Supplement: Supplementary file 2 [file aud-41-289-s002.pdf]

Supplemental Table 2. Hearing: Odds ratios for dietary micronutrients, macronutrients and dietary patterns by quintile adjusting for tinnitus

	Quintile 1	Quintile 2			Quintile 3			Quintile 4			Quintile 5			
	(comparison)	Odds ratio	95% confidence interval		Odds ratio	95% confidence interval		Odds ratio	95% confidence interval		Odds ratio	95% confidence interval		*P* value*
Vitamin B12	-	1.07	0.98	1.17	1.08	0.98	1.19	1.03	0.92	1.14	1.17	1.04	1.31	0.031
Vitamin B6	-	0.91	0.83	1.00	0.93	0.83	1.03	0.94	0.83	1.06	0.99	0.86	1.15	0.181
Vitamin C	-	0.96	0.87	1.05	0.99	0.90	1.09	0.90	0.81	1.00	0.89	0.78	1.00	0.095
Vitamin D	-	1.06	0.97	1.16	0.98	0.89	1.07	0.96	0.87	1.06	0.90	0.81	1.00	0.025
Vitamin E	-	1.02	0.93	1.12	1.04	0.95	1.15	1.07	0.97	1.18	1.14	1.02	1.28	0.197
Carotene	-	1.05	0.96	1.15	1.10	1.00	1.21	1.01	0.92	1.12	1.03	0.92	1.15	0.217
Retinol	-	1.04	0.95	1.14	1.02	0.93	1.12	0.96	0.86	1.06	0.95	0.85	1.07	0.312
Folate	-	0.95	0.87	1.05	0.97	0.87	1.08	0.97	0.87	1.09	0.99	0.87	1.13	0.862
Calcium	-	1.01	0.92	1.11	1.03	0.95	1.12	1.05	0.96	1.15	1.03	0.93	1.14	0.870
Iron	-	0.96	0.87	1.06	0.93	0.84	1.03	0.94	0.84	1.05	0.89	0.79	1.01	0.451
Potassium	-	1.05	0.95	1.15	1.00	0.89	1.12	0.93	0.81	1.05	0.93	0.80	1.09	0.187
Magnesium	-	1.08	0.98	1.20	1.09	0.98	1.22	1.10	0.98	1.25	1.09	0.95	1.26	0.511
Carbohydrate	-	1.01	0.91	1.11	0.96	0.85	1.08	0.96	0.83	1.11	0.88	0.74	1.05	0.353
Fibre	-	1.00	0.91	1.10	1.03	0.92	1.14	1.01	0.90	1.13	0.97	0.84	1.11	0.848
Fat	-	0.95	0.86	1.06	0.99	0.88	1.13	0.96	0.83	1.12	0.98	0.82	1.18	0.808
Saturated Fat	-	1.06	0.96	1.17	1.08	0.96	1.21	1.13	0.99	1.30	1.13	0.95	1.33	0.517
Polyunsat. Fat	-	0.99	0.90	1.08	0.95	0.86	1.05	0.94	0.84	1.05	0.97	0.85	1.11	0.740
Protein	-	0.92	0.84	1.01	0.93	0.84	1.02	0.91	0.82	1.01	0.87	0.78	0.98	0.221
Starch	-	0.99	0.91	1.09	1.04	0.94	1.14	1.07	0.96	1.20	1.08	0.94	1.23	0.603
Sugars	-	1.02	0.92	1.12	1.13	1.01	1.26	1.05	0.92	1.20	1.08	0.92	1.27	0.111
Factor 1 Prudent	-	0.99	0.91	1.08	0.97	0.89	1.05	0.91	0.83	0.99	0.87	0.79	0.95	0.009
Factor 2 High fat	-	1.03	0.94	1.13	1.12	1.02	1.22	1.14	1.04	1.24	1.14	1.04	1.24	0.006
Factor 3 Meat	-	0.99	0.91	1.08	0.99	0.91	1.08	0.95	0.88	1.04	0.93	0.85	1.01	0.411
Factor 4 Sugars & carbohydrates	-	1.03	0.95	1.12	1.06	0.97	1.15	1.04	0.95	1.13	1.02	0.94	1.12	0.769
Factor 5 Polyunsat. fats	-	0.96	0.88	1.04	1.01	0.93	1.10	1.00	0.92	1.09	1.02	0.93	1.11	0.710
Factor 6 Starch	-	1.00	0.92	1.09	1.01	0.92	1.10	1.02	0.94	1.12	1.03	0.94	1.12	0.944

*p value is the statistical significance level for the trend within the regression model.

Nutrient results are multivariable adjusted for age, sex, race, ototoxic medication, BMI, occupation-related noise exposure, smoking status, alcohol consumption, physical activity, hypertension and tinnitus and all other nutrient/dietary factors simultaneously.
